# Mother–Infant Dyadic Neural Synchrony Measured Using EEG Hyperscanning and Validated Using Behavioral Measures

**DOI:** 10.3390/children12020115

**Published:** 2025-01-22

**Authors:** Mary Lauren Neel, Arnaud Jeanvoine, Caitlin P. Kjeldsen, Nathalie L. Maitre

**Affiliations:** 1 Department of Pediatrics, Emory University School of Medicine, Atlanta, GA 30307, USA; caitlin.kjeldsen@emory.edu (C.P.K.); nathalie.linda.maitre@emory.edu (N.L.M.); 2 Department of Pediatrics, Children’s Healthcare of Atlanta, Atlanta, GA 30329, USA; 3 Harmonips LLC, Columbus, OH 43209, USA; arnaudjeanvoine@gmail.com

**Keywords:** hyperscanning, synchrony, parenting, electroencephalography, bondedness, infant

## Abstract

Background/objective: Greater parent–infant synchrony is associated with improved child outcomes. Behavioral measures of synchrony are still developing in young infants; thus, researchers need tools to quantify synchrony between parents and their young infants. We examined parent–infant neural synchrony measured using dual EEG hyperscanning and associations between neural synchrony, infant behavioral measures of synchrony, and maternal bondedness and depression. Methods: Our prospective cohort study included mother–infant dyads at 2–4 months of age. We collected time-locked dual EEG recordings of mother and infant and simultaneous video-recordings during a scaffolded interaction where the mother sequentially layered sensory modalities to the interaction. Neural synchrony measured using EEG hyperscanning was analyzed using the circular correlation coefficient (CCorr), infant behavioral synchrony was measured using the validated Welch Emotional Connection Screen (WECS) scores, and maternal bondedness and depression were measured using standardized questionnaires. Results: Our study included n = 47 dyads. Dyadic CCorr increased across the interaction as the mother added tactile stimulation to visual stimulation. We also found associations between behavioral and neural measures of dyadic synchrony such that infants with higher scores on behavioral measures of emotional connection on the WECS showed greater increases in CCorr indicative of dyadic synchrony with their mother across this interaction. We found no associations between neural synchrony and maternal bondedness or depression. Conclusion: These findings support the construct validity of mother–infant dyadic neural synchrony measured using EEG hyperscanning and analyzed using CCorr. Opportunities for future research on quantification of neural synchrony between parents and young infants abound.

## 1. Introduction

Parent–infant interactions are arguably the most influential factor in early child development [1,2,3,4]. Parent–infant synchrony is defined as time-bound co-regulation between parent and child [5]. Studies have shown that greater parent–infant synchrony is associated with improved child cognitive and behavioral outcomes [5,6,7,8,9]. Parent–infant synchrony is modifiable [10,11,12,13], presenting an opportunity to promote child development [9,14]; however, quantifying parent–infant synchrony presents a challenge. Although behavioral measures are valid, they have limitations in the early months of life as facial expressions are still developing [15,16,17,18]. Thus, researchers need psychometrically sound neural measures to quantify parent–infant synchrony in young infants before consistent synchrony behaviors are established. Such neural measures could serve as biomarkers of at-risk dyads and response to intervention.

Currently, neural measures of synchrony also have limitations. While some studies use fMRI to measure neural synchrony [19], this is expensive and necessitates an immobile participant and tolerance of loud machinery noises, making its use challenging for young children [20,21]. Functional near-infrared spectroscopy (fNIRS) has also been used to measure synchrony [20]; however, it has unique challenges in preterm infants due to the variability of cerebral blood flow and, in general, has limited temporal resolution compared to other modalities such as electroencephalography (EEG) [21,22,23].

The recent literature has suggested that dual EEG hyperscanning can be used to quantify interpersonal neural synchrony [9,14,24] even in young infants and their parents. circular correlation coefficient (CCorr) is the most promising measure of neural synchrony due to its robustness to spurious measurement errors [24,25]. Although few studies assess construct validity between behavioral and neural measures in infants [22,26,27], none use neural measures analyzed using CCorr. To our knowledge, ours is the first study to assess neural synchrony measured using CCorr and behavioral measures of synchrony during a parent–infant interaction designed to elicit increased synchrony between the parent and infant over time.

Therefore, we designed a study to test our hypothesis that mother–infant neural synchrony as measured using dual EEG hyperscanning and analyzed using CCorr would increase across a scaffolded interaction where the mother sequentially layers additional sensory modalities to the interaction. We also explored associations between mother–infant neural synchrony and infant behavioral measures indicative of synchrony, as well as between mother–infant neural synchrony and maternal bondedness/depression scores. We hypothesized that neural measures of synchrony would positively correlate with infant behavioral measures and maternal bondedness and inversely correlate with maternal depression.

## 2. Materials and Methods

### 2.1. Participants

This prospective observational study included healthy infants without birth complications born after ≥34 weeks of gestation who were 2–4 months corrected age (CA) at the time of the study. The exclusion criteria were as follows: infants exhibiting vision or hearing abnormalities, those unable to visually track a 10-degree deviation from the midline in either direction, or those with direct or maternal exposure to analgesics or sedatives within 72 h prior to the study procedures. The study received approval from the hospital’s Institutional Review Board (IRB 17-00559), and written informed consent was obtained from the parent(s) of each infant. Data were collected between July and November 2017 in our clinical research laboratory setting. The methodology is described in detail in our previous publication [28]. Sixty dyads consented to and completed our study protocol.

### 2.2. Procedures

**Figure 1:**
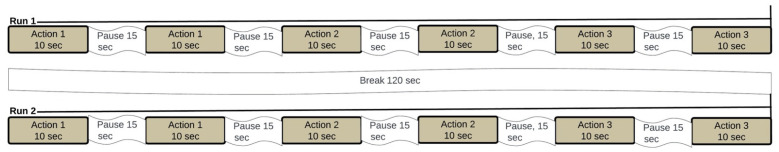
Sequence of scaffolded multisensory interactions for hyperscanning analysis of mother–infant neural synchrony. Action 1: The mother smiles at the infant (visual stimulus); Action 2: The mother smiles and touches the infant’s cheek (visual + tactile stimuli); Action 3: The mother smiles, touches the infant’s cheek, and verbally expresses with emotion, “I love you, [baby’s name]” (visual + tactile + auditory stimuli). Each subsequent action introduces an additional layer of maternally provided sensory scaffolding [28].

We collected time-locked dual EEG recordings of mother and infant during the interaction using simultaneous video recording. Infant behaviors on video recording were subsequently coded using the Welch Emotional Connection Screen [28,29]. We did not code maternal behaviors given that they were scripted by the interaction. During the interaction, mother–infant dyads engaged in a structured naturalistic interaction in which the mother provided infant-directed sensory stimuli [28]. The mother performed a series of three distinct actions, each repeated twice, with the actions sequenced to progressively enhance sensory scaffolding between mother and infant (Figure 1). These actions were designed to foster mother–infant engagement.

As detailed in a previous study [28], mothers received scripted instructions for performing each action and were monitored by an examiner to ensure procedural fidelity. Each individual action lasted for 10 s, followed by a 15 s pause to minimize anticipation or habituation effects. A complete sequence, or “run”, consisted of six actions, repeated twice with a two-minute break between runs (Figure 1). The mother and infant were positioned across from one another, with their faces placed at the optimal focusing distance for the infant (24–30 inches). An examiner, positioned behind the infant, provided cues to the mother regarding the initiation and conclusion of each action [28]. The EEG computer and operator were in the room but outside of the infant’s field of vision. All other personnel observed from behind a two-way mirror in an adjacent observation room. In Action 1, the mother smiles at the infant; in Action 2, the mother smiles and gently touches the infant’s cheek; and in Action 3, the mother smiles, touches the infant’s cheek, and verbally expresses with emotion, “I love you, [baby’s name]” (Figure 1).

### 2.3. Electroencephalography Hyperscanning and Circular Correlation Coefficient as a Neural Measure of Synchrony

Electroencephalography (EEG) was continuously recorded during the multisensory interaction using a high-density 128-electrode array embedded in soft sponges, fitted to appropriately sized caps for both the mother and the infant (Hydrocel Sensor Net, EGI, Inc., Eugene, OR, USA). The reference electrode was placed at the vertex, and data were collected at a sampling rate of 1000 Hz (Net Station v. 5.2.0.2; EGI, Inc., Eugene, OR, USA). An auditory countdown cue was used to signal transitions between actions and to mark the corresponding EEG periods with a manual trigger [7]. These markers were further validated by a concurrent video recording [28]. Pauses between actions, intended to prevent anticipation or habituation, were segmented and excluded from the EEG analyses.

Impedance was verified to be <50 kΩ at all electrodes prior to the commencement of the paradigm to minimize the occurrence of unusable electrode data. EEG data were processed using Net Station software v4.3. The recorded signal underwent preprocessing, which included the application of a 60 Hz notch filter and a 0.3–40 Hz bandpass filter (Butterworth, order = 1) [30]. Amplitudes exceeding 120 µV were excluded to mitigate artifacts, including eye blinks and movement. The EEG signal was then re-referenced to the average reference. Action segments were excluded if more than 35 electrodes exhibited unusable data during the recording. For subjects with fewer than 35 unusable electrodes, noisy data were corrected through interpolation using surrounding electrodes and standard algorithms in Net Station [28].

We used the 6–9 Hz alpha band to analyze mother–infant CCorr, based on previous reports and best evidence [31,32]. Analyses focused on differences in the circular correlation coefficient (CCorr) between the three actions. CCorr was calculated using the following equation:

$$CCorr={\left|\frac{\sum_{i=1}^{n}\sum_{j=1}^{n}\sin\left(ai-\bar{a}\right)sin(bj-\bar{b})}{\sqrt{\sum_{i=1}^{n}{sin(ai-\bar{a})}^{2}\sum_{j=1}^{n}{sin(bj-\bar{b})}^{2}}}\right|}^{2}$$

where $\left(ai-\bar{a}\right)$ is the circular phase for babies and $\left(bi-\bar{b}\right)$ is the circular phase for moms. CCorr values are between 0 and 1. The CCorr formula was scripted in Python 2.6 [33] and NumPy 2.2.0 [34,35].

## 3. Additional Measures

### 3.1. Welch Emotional Connection Scale (WECS)

As outlined in a previous publication [28], infant behavioral responses to each action were coded using a standardized three-point scale from WECS [28,29]. The coding encompassed three domains: facial expressiveness, sensitivity to the mother (i.e., responsiveness to the mother’s expressed emotions), and vocal communication [29]. Infant responses were scored as follows: 1 for a negative response, 2 for a variable or mixed response, and 3 for a positive response, based on standardized coding criteria outlined in the literature [28]. The Welch Emotional Connection Scale (WECS) has demonstrated strong validation as an indicator of mother–infant behavioral responsiveness [29,36], with higher scores reflecting a stronger emotional connection to the mother. Raw scores were averaged across the four iterations of each action to compute final scores for each domain and action. Inter-rater reliability was established at >85% among three coders for all infant behavioral domains, assessed on a subset of 10 videos. Video recordings were then randomly allocated among the three coders, with the primary coder reviewing over 50% of the videos as a secondary coder to maintain consistency and ensure inter-rater reliability.

### 3.2. Mother-to-Infant Bonding Scale (MIBS)

To assess the maternal perception of bondedness, we used the Mother-to-Infant Bonding Scale (MIBS) [37]. The MIBS is an eight-item questionnaire quantifying to what extent an adjective describes the mother’s feelings towards their child. The score range is 0–24, with a lower score being indicative of a higher perception of bonding. The at-risk threshold is ≥2 [37,38,39]. Cronbach’s alpha is acceptable at 0.71 [37,39].

### 3.3. Edinburgh Postnatal Depression Screen (EPDS)

To quantify maternal risk for postnatal depression, we used the Edinburgh Postnatal Depression Screen (EPDS) [40]. The EPDS is a 10-item questionnaire. The score range is 0–30, with a higher score being indicative of a greater risk of depression. The at-risk threshold is conservatively ≥10 [37,38,40]. Cronbach’s alpha is good at 0.87 [40].

Both the MIBS and EPDS are highly feasible and well-established measures [37,38].

### 3.4. Statistical Analyses

We used repeated measures analysis of variance to determine if there were differences between CCorr across actions. We then used follow-up contrasts adjusting for multiple comparisons using Bonferroni correction to control for alpha based upon three contracts, given the three actions. Exploratory analyses of change in CCorr over time and associations with infant WECS (behavioral) scores and maternal bondedness/depression were conducted using general linear models with repeated measures.

## 4. Results

Our final cohort included n = 47 mother–infant dyads (Table 1). Although a total of 60 dyads were initially enrolled, n = 13 were excluded from the analysis due to poor-quality or missing EEG data for either the mother or the infant, or technical issues with the recording (e.g., trigger errors). The final sample included 47 dyads. The median gestational age was 39 weeks, and the median age at testing was 3 months. The majority of participants identified as White and non-Hispanic.

**Figure 2:**
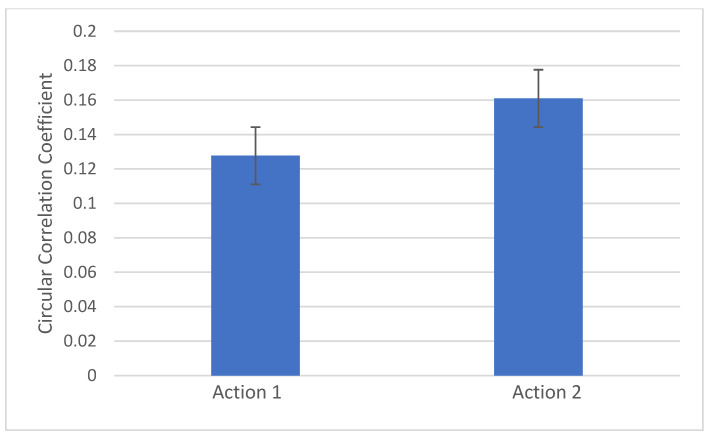
Parent–infant neural synchrony increases between Actions 1 and 2. Mother–infant CCorr increased significantly between Action 1 (visual) and Action 2 (visual + tactile) (*p* = 0.015).

Repeated measures analysis of variance revealed differences between CCorr across actions (F (2,92) = 3.5, *p* = 0.034). Follow-up contrasts adjusted for multiple comparisons using Bonferroni correction revealed significantly higher neural synchrony during Action 2 compared to Action 1 (*p* = 0.015). Dyadic CCorr increases significantly between Action 1 (visual input) and Action 2 (visual + tactile) (Figure 1 and Figure 2). There were no significant differences between Actions 1 or 2 and Action 3 (visual + tactile + auditory) (*p* > 0.017).

Our previous paper reported on the increase in infant WECS (behavioral) scores across the interaction as the mother added further sensory stimuli to the actions [28]. Similar to that analysis, we found progressive increases in WECS scores for infant facial expressiveness across actions (WECS facial in Action 1 < Action 2 < Action 3) with significant differences between Actions 1 and 3 (*p* = 0.002) and Actions 2 and 3 (*p* = 0.007) (Supplementary Figure S1).

Upon exploratory analyses, we found significant differences in dyadic CCorr over time associated with infant WECS (behavioral) scores. Infants with higher WECS scores for facial expressiveness demonstrated greater increases in dyadic CCorr between Actions 1 and 2 as compared to those with lower scores (*p* = 0.031). Similarly, infants with higher WECS scores for vocal communication demonstrated greater increases in dyadic CCorr between Actions 1 and 2 as compared to those with lower scores (*p* = 0.007). We did not find significant associations between CCorr over time and infant sensitivity to parent WECS scores (Table 2).

Similarly, we found that infants with higher WECS scores for infant sensitivity to parents demonstrated greater increases in dyadic CCorr between Actions 1 and 3 as compared to those with lower scores (*p* = 0.043). Infants with higher WECS scores for facial expressiveness and vocal communication also showed greater increases in dyadic CCorr between Actions 1 and 3 as compared to those with lower scores. However, these levels only approached significance (*p* = 0.094, *p* = 0.08, respectively) (Table 3).

We found no significant differences in CCorr between Actions 2 and 3 with any WECS measures. No parents met the criteria for depression, so we did not run further analyses with this variable. There were no significant differences in CCorr between Actions 1, 2, or 3 associated with the maternal perception of bondedness.

## 5. Discussion

CCorr, a measure of neural synchrony obtained using EEG hyperscanning, increases across a dyadic interaction as the mother adds additional sensory input (from Action 1 to Action 2). In our paradigm, the addition of maternal touch was associated with significant increases in dyadic CCorr. Previous work has suggested the salience of affective maternal touch for synchrony [27], and has specifically linked touch between mother and 4–6-month-old infant with neural synchrony on fNIRS [41].

The addition of maternal voice to touch did not increase CCorr in our paradigm. While it is possible that maternal touch promotes dyadic synchrony to a greater degree than does maternal voice, it is perhaps more likely that this resulted from touch being the first multisensory condition (in this case, visual + tactile) and therefore triggering dyadic synchrony [9,28]. To compare the salience of tactile versus auditory stimulation in promoting dyadic synchrony, the order of the stimuli would have needed to be randomized, which was outside the scope of this study. Infant-directed speech (IDS) has been associated with infant neural responsiveness on NIRS, specifically in the prefrontal cortex [42] and temporal regions [43] for non-parent IDS and frontal regions for parent IDS [43]. In addition, infants demonstrate different cortical activation patterns in the EEG power spectral density in response to maternal IDS as compared to maternal adult-directed speech [44]. While IDS has been hypothesized to promote mother–infant synchrony [25,27], this has not yet been evaluated with dyadic hyperscanning.

This study answers the call in the literature for the validation of neural synchrony measures with established behavioral measures [22,26]. In this study, we found associations between behavioral and neural measures of dyadic synchrony: infants with higher behavioral measures of emotional connection on the validated WECS [29] showed greater increases in CCorr indicative of dyadic synchrony with their mother across this interaction. These findings support the construct validity of neural synchrony measured using EEG hyperscanning and CCorr with infant behaviors in the WECS.

Our previous work has shown infant WECS scores and infant neural measures of responsiveness, quantified using the EEG Frontal Asymmetry Score (FAS), increase across the actions in this paradigm [28]. However, that work assessed only infant behavior and neural measures rather than dyadic neural measures. Previous studies by other groups have demonstrated that parent and child behavior and neural responsivity are connected; specifically, that parental behavior impacts child neural responsivity and vice versa [25,45]. In one study, parental neural responsivity to the child, measured using EEG theta power, was associated with increased infant sustained attention [45]. While this is not dyadic EEG or hyperscanning, it does demonstrate separate associations between parent and child neural and behavioral responsivity for both parent and child.

Interestingly, we did not find associations between dyadic neural synchrony and maternal perceptions of bondedness. Our previous work with this interaction paradigm revealed that infants whose mothers scored high for bondedness showed increasing WECS vocal communication across actions (as compared to infants whose mothers scored low for bondedness); however, infant neural responsiveness (as measured using FAS) was not different between the high and low maternal bondedness groups [28]. Because the Mother-to-Infant-Bonding Scale [37] is a measure of the maternal perception of bondedness, this may or may not reflect the infant experience. Previous work has linked maternal perceptions of bondedness to maternal neural physiological markers, such as greater maternal fMRI activation patterns in the middle frontal gyrus upon viewing her own infant versus another infant [46]; however, this is a neural measure of mother responsiveness, not dyadic synchrony. To our knowledge, this is the first study to examine dyadic EEG hyperscanning measures of synchrony with maternal perceptions of bondedness. Further work is needed to explore the complex relationships between the maternal perception of bondedness, infant experience of bondedness or attachment to mother, and measures of dyadic neural synchrony.

## 6. Limitations

Though the WECS is a dyadic measure of emotional connection, we were only able to use the infant scores and not the mothers’, since the maternal portion of the interaction was scripted. Future work could study a less scripted interaction to allow for the use of dyadic behavioral scores. In addition, we only included mothers for this study to minimize confounding factors, but future, larger studies should include fathers and/or other caregivers.

We did not randomize the order of stimuli presentation, limiting our ability to draw conclusions about tactile versus auditory stimulation, as this was outside our study’s scope. Our study population was primarily White and non-Hispanic; future work should confirm that these findings generalize to more diverse samples. Finally, this study only describes a one-time visit using repeated measures; future work could examine longitudinal dyadic synchrony over multiple study visits.

## 7. Conclusions

CCorr, a measure of neural synchrony obtained using EEG hyperscanning, increases across a dyadic interaction as the mother progressively adds additional sensory input to the interaction. Associations between behavioral and neural measures of dyadic synchrony revealed that infants with higher scores on behavioral measures of emotional connection on the WECS showed greater increases in CCorr indicative of dyadic synchrony with their mother across this interaction. These findings support the construct validity of dyadic neural synchrony using EEG hyperscanning analyzed using CCorr. Such methodologies to assess early dyadic synchrony could be used to identify at-risk dyads or to quantify dyadic response to parenting interventions in infancy or early childhood to ensure these interventions achieve maximal impact.

## Figures and Tables

**Table 1:** Population characteristics and scores.

Infant Data (N = 47)	N, Unless Noted *	%, Unless Noted *
Female	24	51
Gestational age in weeks (median, IQR) *	39	(39, 40)
Corrected Age in days (median, IQR) *	94	(78, 114)
Race		
White	36	77
Black or African-American	2	4
Asian	2	4
More than one race	7	15
Ethnicity		
Hispanic	3	6
Not Hispanic	41	87
No response	3	6
WECS facial expressiveness (median, IQR) *	1.7	(1.4, 2.0)
WECS sensitivity to parent (median, IQR) *	2.5	(2.2, 2.8)
WECS vocal communication (median, IQR)*	2.0	(1.9, 2.2)
**Maternal Data (N = 47)**		
Maternal Education		
Partial College or Trade School	6	13
College Graduation	15	32
Graduate Education	26	55
MIBS Score (median, IQR) *	1	(0, 2)
EPDS Score (median, IQR) *	3	(1, 5)

* EPDS: Edinburgh Postnatal Depression Scale; MIBS: Mother-to-Infant Bonding Scale; WECS: Welch Emotional Connection Screen.

**Table 2:** Dyadic circular correlation coefficient increases more across the interaction (between Actions 1 and 2) for infants with higher WECS scores.

	WECS Facial 1	WECS Facial 2/3
Difference between CCorr Action 2–Action 1 *	0.012	0.096
	**WECS Vocal 1**	**WECS Vocal 2/3**
Difference between CCorr Action 2–Action 1 *	0.008	0.038

Dyadic CCorr (neural synchrony) increases more across interaction for infants with higher WECS scores for facial expressiveness (*p* = 0.031) and vocal communication (*p* = 0.007). This suggests construct validity for neural measure of synchrony with established behavioral measure of emotional connection/synchrony. * *p* < 0.05.

**Table 3:** Dyadic circular correlation coefficient increases more across the interaction (between Actions 1 and 3) for infants with higher WECS scores.

	WECS sensitivity 1	WECS sensitivity 2/3
Difference between CCorr Action 3–Action 1 *	−0.072	0.019
	**WECS facial 1**	**WECS facial 2/3**
Difference between CCorr Action 3–Action 1	−0.023	0.031
	**WECS vocal 1**	**WECS vocal 2/3**
Difference between CCorr Action 3–Action 1	−0.003	0.056

Dyadic CCorr (neural synchrony) increases more across interaction for infants with higher WECS scores for sensitivity to parents (*p* = 0.043). This suggests construct validity for neural measure of synchrony with established behavioral measure of emotional connection/synchrony. * *p* < 0.05.

## Data Availability

The datasets generated and analyzed during the current study are not publicly available due to them containing protected health information, but are available from the corresponding author upon reasonable request from the scientific community.

## Supplementary Materials

The following supporting information can be downloaded at: https://www.mdpi.com/article/10.3390/children12020115/s1, Figure S1: Baby facial expressiveness scores on Welch Emotional Connection Screen (WECS) increase across the interaction. Infant facial expressiveness scores on WECS increased significantly between Action 1 (visual) and Action 3 (visual + tactile + auditory) (*p* = 0.002) and between Action 2 (visual + tactile) and Action 3 (visual + tactile + auditory) (p=0.007). There were no significant changes in WECS scores for infant sensitivity to parent and infant vocal communication across actions.
