# Supplementary material for: Mother–Infant Dyadic Neural Synchrony Measured Using EEG Hyperscanning and Validated Using Behavioral Measures
Source: Children (Basel). 2025 Jan 22;12(2):115. doi: 10.3390/children12020115 (PMC11854398; doi:10.3390/children12020115)
Supplement: Supplementary file 1 [file children-12-00115-s001.zip › children-3397000-supplementary.pdf]

Supplementary information:

Supplementary Figure 1: Baby facial expressiveness scores on Welch Emotional Connection Screen (WECS) increase across the interaction

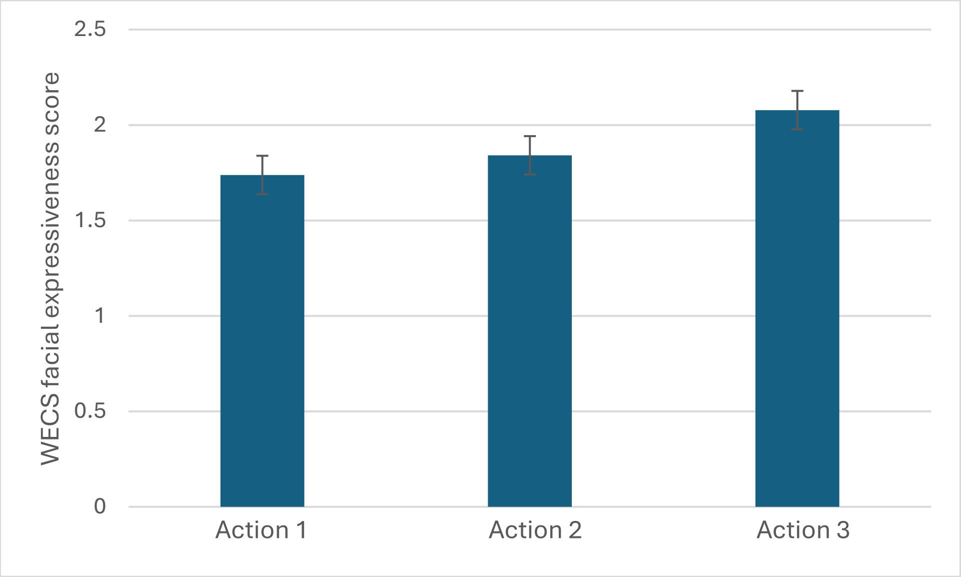

Caption: Infant facial expressiveness scores on WECS increased significantly between Action 1 (visual) and Action 3 (visual + tactile + auditory) (p=0.002) and between Action 2 (visual + tactile) and Action 3 (visual + tactile + auditory) (p=0.007). There were no significant changes in WECS scores for infant sensitivity to parent and infant vocal communication across actions.
